# Neuromuscular Control Deficits After Anterior Cruciate Ligament Reconstruction: A Pilot Study Using Single-Leg Functional Tests and Electromyography

**DOI:** 10.3390/jfmk10010098

**Published:** 2025-03-19

**Authors:** Ayrton Moiroux--Sahraoui, Jean Mazeas, Maxime Gold, Georgios Kakavas, Florian Forelli

**Affiliations:** 1 Orthosport Rehab Center, 95330 Domont, France; ayrton.moirouxsahraoui@gmail.com (A.M.--S.); jeanmazeas@gmail.com (J.M.); 2 Orthopaedic Surgery Department, Clinic of Domont, Ramsay Healthcare, @OrthoLab, 95330 Domont, France; orthosport.kine@gmail.com; 3 Fysiotek Spine & Sports Lab, 116 35 Athens, Greece; georgios.kakavas@gmail.com; 4 Department of Physical Education and Sport Sciences, ErgoMech-Lab, University of Thessaly, 421 00 Volos, Greece; 5 SFMK Lab, 93380 Pierrefite sur Seine, France; 6 Haute-Ecole Arc Santé, HES-SO University of Applied Sciences and Arts Western Switzerland, 2000 Neuchâtel, Switzerland

**Keywords:** ACL reconstruction, neuromuscular control, gluteus medius, vastus medialis, surface electromyography

## Abstract

**Purpose:** This study aimed to evaluate neuromuscular control and muscle activation patterns in individuals following anterior cruciate ligament (ACL) reconstruction, compared to healthy controls. **Methods**: A cross-sectional comparative study was conducted following STROBE guidelines, including 16 participants (ACL group: n = 9; control group: n = 7). Participants performed the single-leg squat (SLS) test and the single-leg drop landing (SLDL) test. Neuromuscular control was assessed using the Qualitative Analysis of Single-Leg Loading Score (QASLS), while gluteus medius and vastus medialis activation were recorded using surface electromyography. **Results**: The ACL group showed significantly higher QASLSs in the SLS test (*p* = 0.0113), indicating poorer movement quality, while no difference was found in the SLDL test (*p* = 0.5484). Gluteus medius activation was lower in the ACL group during the SLS test (*p* = 0.0564), and vastus medialis activation was higher but not significantly different (*p* = 0.095). **Conclusions:** These findings highlight persistent neuromuscular deficits post-ACL-reconstruction, particularly in SLS tasks, reinforcing the need for targeted rehabilitation strategies focusing on hip stabilization and quadriceps motor control to optimize movement quality and reduce reinjury risk.

## 1. Introduction

Anterior cruciate ligament (ACL) injuries are among the most common and serious musculoskeletal injuries in athletes, with an estimated incidence of 200,000 to 250,000 cases per year in the United States alone [1,2]. ACL tears typically occur through non-contact mechanisms, often involving sudden deceleration, pivoting, or landing from a jump with improper knee alignment [3,4]. The biomechanical pattern most frequently associated with non-contact ACL injuries is dynamic knee valgus, characterized by excessive medial knee displacement, femoral internal rotation, and tibial external rotation [5,6,7]. This movement pattern places significant strain on the ACL, increasing the likelihood of rupture.

Beyond the immediate consequences of ACL injury, including pain, functional limitations, and a prolonged rehabilitation process, long-term complications such as post-traumatic osteoarthritis, decreased neuromuscular control, and an increased risk of reinjury are well-documented [8,9,10]. Even after surgical reconstruction, many athletes struggle to regain full knee function and return to pre-injury performance levels. Studies indicate that up to 45% of athletes fail to return to their prior level of sports participation, with psychological factors such as fear of reinjury and biomechanical deficits contributing to this limitation [11,12,13].

One of the key factors influencing both injury risk and post-reconstruction rehabilitation is neuromuscular control, which refers to the ability of the central nervous system to coordinate muscle activation patterns to stabilize the knee joint [14]. Impairments in neuromuscular control following ACL reconstruction can result in altered movement patterns, compensatory strategies, and an increased likelihood of secondary injuries. Studies suggest that these deficits may be linked to muscle imbalances between the hip and quadriceps muscles, which play a crucial role in knee joint stabilization during functional activities [15,16,17]. The vastus medialis plays a crucial role in patellar tracking and knee joint stability, with altered activation patterns often observed in individuals following ACL reconstruction [9,12]. Deficits in vastus medialis function have been linked to quadriceps inhibition and compensatory movement strategies, which can impact functional recovery and reinjury risk.

The gluteus medius and vastus medialis play a key role in knee joint stabilization, neuromuscular control, and ACL injury prevention. Several studies have demonstrated that deficits in the activation of these muscles contribute to abnormal movement patterns, increased ACL injury risk, and impaired recovery following ACL reconstruction [18,19,20,21]. Their functions are particularly relevant during dynamic, weight-bearing tasks, such as single-leg squats (SLS) and single-leg drop landings (SLDLs), where proper activation is crucial for joint stability and optimal force distribution.

The gluteus medius plays a pivotal role in controlling frontal-plane stability, limiting excessive hip adduction, and reducing dynamic knee valgus, which is a well-documented risk factor for ACL injury [22,23]. Research indicates that insufficient activation or delayed recruitment of the gluteus medius leads to increased medial knee displacement, placing greater strain on the ACL during cutting, landing, and pivoting maneuvers [24,25]. This phenomenon is particularly problematic in ACL-reconstructed individuals, as compensatory strategies involving increased trunk lean and hip internal rotation are commonly observed during rehabilitation [20,21,26].

Numerous studies have assessed gluteus medius activation patterns during functional tasks to evaluate its contribution to neuromuscular control. For instance, Stearns and Powers demonstrated that gluteus medius activation deficits were associated with abnormal knee mechanics during single-leg landings, potentially predisposing athletes to secondary ACL injuries [23]. Similarly, Hollman et al. found that asymmetrical gluteus medius function resulted in greater knee valgus angles, reinforcing the importance of the hip musculature in lower-limb alignment [24]. Given these findings, assessing gluteus medius activation during SLS and SLDL tasks provides valuable insight into neuromuscular deficiencies post-ACL-reconstruction and aids in identifying high-risk movement patterns.

The vastus medialis is essential for patellar tracking, knee joint alignment, and quadriceps force production [27,28]. Following ACL reconstruction, quadriceps dysfunction and neuromuscular inhibition are commonly observed, often leading to compensatory movement strategies and altered lower-limb biomechanics [29,30]. Insufficient activation of the vastus medialis has been linked to reduced knee extension strength, a greater reliance on passive joint structures, and poor dynamic control during functional movements [31].

Studies using sEMG to assess vastus medialis activation post-ACL-reconstruction have reported persistent activation delays, lower muscle recruitment, and asymmetrical force distribution, even after patients meet return-to-sport criteria [28,31,32,33]. Dingenen et al. found that reduced vastus medialis activation was correlated with compensatory hip strategies, further supporting the interplay between quadriceps and hip stabilizers in neuromuscular control [34]. Additionally, Paterno et al. identified vastus medialis asymmetries as a predictor of movement deficiencies during dynamic tasks, reinforcing its relevance in ACL rehabilitation research [29].

The Qualitative Analysis of Single-Leg Loading Score (QASLS) was selected as the primary movement quality assessment tool in this study due to its validated ability to detect compensatory movement patterns in ACL-reconstructed individuals [30]. Traditional neuromuscular assessments such as isokinetic strength testing and joint position sense analysis provide valuable quantitative measures of force production and proprioception but fail to capture real-world movement deficiencies [28,35]. In contrast, QASLS enables the assessment of dynamic movement patterns, including trunk stability, knee valgus, and postural control, which are critical components of return-to-sport evaluations [26].

Previous studies have demonstrated that qualitative movement assessments like QASLS can effectively identify high-risk biomechanical patterns post-ACL-reconstruction [29]. For instance, studies have shown that persistent knee valgus and asymmetrical movement patterns during single-leg tasks correlate with a higher risk of reinjury [36,37]. Moreover, the clinical applicability of QASLS makes it more accessible for rehabilitation professionals compared to motion capture or force-plate analyses, which require specialized equipment [34]. Given that movement quality impairments may persist even in individuals who meet return-to-sport strength criteria, QASLS serves as a functional and practical tool for evaluating neuromuscular deficits beyond strength alone.

The aim of this study is to compare neuromuscular control and the activation of the gluteus medius and vastus medialis muscles during two functional tests: the single-leg squat (SLS) and the single-leg drop landing (SLDL). Specifically, we seek to determine whether individuals who have undergone ACL reconstruction exhibit differences in muscle activation patterns and movement quality compared to healthy individuals. This research will contribute to a better understanding of post-ACL-reconstruction motor control deficits and may inform rehabilitation strategies aimed at reducing reinjury risk.

## 2. Materials and Methods

### 2.1. Study Design

This study followed a cross-sectional comparative design to evaluate neuromuscular control and muscle activation patterns in individuals who had undergone ACL reconstruction, compared to healthy controls.

This study was conducted in accordance with the STROBE (Strengthening the Reporting of Observational Studies in Epidemiology) guidelines, ensuring methodological rigor and transparent reporting of observational research [38]. Ethical approval was obtained following the principles of the Declaration of Helsinki (World Medical Association, 2013). Each participant provided written informed consent before enrollment in the study.

### 2.2. Participants

This study included 16 participants, divided into two groups: the ACL group (9 individuals with a history of ACL reconstruction) and the control group (7 healthy individuals with no prior knee injuries).

Participants in the ACL group were between 18 and 40 years old and had undergone ACL reconstruction at least six months before the study. They were required to have returned to at least moderate physical activity and had their surgery using a standardized technique (hamstring tendon or patellar tendon autograft).

We excluded individuals who had severe post-operative complications, such as graft failure or chronic knee instability. Participants with neuromuscular disorders or those who had undergone bilateral ACL surgeries were also excluded.

The control group consisted of healthy individuals with no history of ACL injuries, lower-limb surgeries, or musculoskeletal disorders. They had no lower-limb injuries in the past six months and no neuromuscular impairments. All participants were engaged in regular physical activity.

To ensure a fair comparison, we matched the control group to the ACL group by age, sex, and physical activity level. Participants were recruited from local sports clubs, physiotherapy clinics, and university athletic programs. Before selection, all individuals completed a screening questionnaire to confirm that they met the inclusion criteria.

This detailed selection process ensured that both groups were comparable and that the study results were reliable.

### 2.3. Rehabilitation Protocol

All individuals in the ACL group followed a standardized post-surgical rehabilitation program, based on international recommendations for ACL recovery [30,39]. The rehabilitation process was divided into three main phases.

During the early post-operative phase (weeks 1 to 6), the focus was on pain management, swelling reduction, and restoring the knee range of motion. Patients engaged in quadriceps activation exercises, including electrical stimulation and straight-leg raises, while avoiding excessive loading of the knee joint.

In the strengthening and neuromuscular re-education phase (weeks 7 to 16), participants engaged in progressive resistance training, incorporating closed-chain exercises such as squats and lunges to enhance quadriceps and hamstring strength [40,41]. Additionally, proprioceptive training was introduced, involving balance exercises on unstable surfaces to improve joint stability. Particular emphasis was placed on gluteus medius strengthening, with exercises such as side-lying hip abductions and resistance-band lateral walks to improve hip control and reduce knee valgus.

The final return-to-sport phase (weeks 16 to 24 and beyond) aimed at restoring functional performance and movement efficiency. Training included plyometric drills, such as box jumps and single-leg drop landings, as well as sport-specific movement retraining [42]. Clearance for return to sport was based on established objective criteria, including an isokinetic strength symmetry of at least 90%, successful completion of functional hop tests, and psychological-readiness assessments using the ACL Return to Sport after Injury scale.

### 2.4. Assessment Protocol

To evaluate neuromuscular control and lower-limb muscle activation, all participants underwent functional movement assessments and sEMG recordings during two key single-leg tasks. These tests were chosen for their relevance in ACL rehabilitation and their ability to assess dynamic knee stability and movement coordination [13,25,30,43].

**Figure 1:**
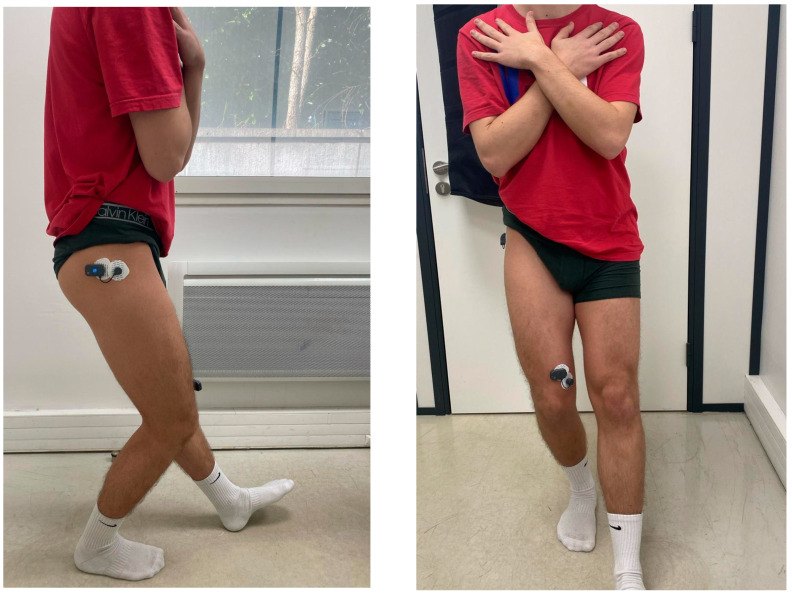
Lateral and Front Views of the Single-Leg Squat.

The SLS test was performed with participants standing on a single leg while squatting to approximately 60° of knee flexion, maintaining trunk stability and proper knee alignment. Movement quality was analyzed using video recordings, with a particular focus on knee valgus and overall postural control (Figure 1).

**Figure 2:**
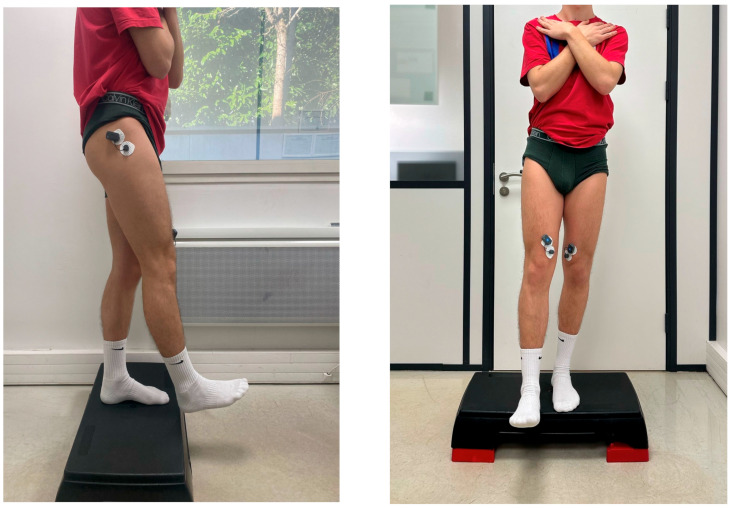
Lateral and Front Views of the Single-Leg Drop Landing.

The SLDL test required participants to step off a 15 cm platform and land on their reconstructed or dominant leg, simulating real-world sports movements. Landing mechanics were assessed, specifically evaluating knee valgus angles, stabilization time, and trunk alignment (Figure 2).

### 2.5. Neuromuscular Control Analysis

To assess neuromuscular control, the Qualitative Analysis of Single-Leg Loading Score (QASLS) was used. This validated scoring system evaluates six movement criteria, including trunk stability, knee valgus, and hip control [30]. Higher QASLSs indicate poorer movement quality, highlighting deficits in dynamic knee stability and motor control.

#### 2.5.1. Surface Electromyography Analysis

Muscle activation patterns were recorded using wireless surface electromyography (sEMG) equipment (FREEEMG, BTS Bioengineering^®^, Milan, Italy), following the international recommendations established by the Surface Electromyography for the Non-Invasive Assessment of Muscles (SENIAM) guidelines. To minimize signal contamination and ensure optimal acquisition, electrode placement was standardized to avoid overlap with innervation zones and reduce cross-talk from surrounding muscles.

For the gluteus medius, electrodes were positioned at 50% of the distance between the iliac crest and the greater trochanter, aligned parallel to the muscle fibers, ensuring consistent and reproducible recordings. For the vastus medialis, electrodes were placed at 80% of the line between the patella and the anterior superior iliac spine, in accordance with SENIAM recommendations [44]. The skin preparation protocol included shaving, light abrasion, and cleaning with alcohol to reduce impedance and improve signal quality [45,46].

Raw EMG signals were captured using a biological amplifier (CMRR > 95 dB), high input impedance (10 MΩ), and low noise levels (<5 μV RMS). The signals were processed using EMG Analyzer software Version 2.9 (BTS Bioengineering^®^, Milan, Italy), where centering, smoothing, and a 50-value moving average filter were applied to eliminate artifacts and optimize signal quality. Normalization to Maximal Voluntary Isometric Contraction (MVIC) values were obtained to allow for accurate comparison of activation levels between participants and tasks.

#### 2.5.2. Maximal Voluntary Isometric Contraction

To ensure data consistency, electromyographic signals were normalized to each participant’s MVIC. The MVIC test consisted of a five-second maximal contraction for both the quadriceps and gluteus medius muscles, with the peak activation value used for normalization. This procedure allowed for a direct comparison of muscle activation levels across different participants and tasks.

Quadriceps MVIC measurement: Participants were seated on a stable surface, with hips and knees flexed at 90 degrees. A strap was placed around the ankle to provide resistance. They were instructed to perform a maximal isometric knee extension against the strap for five seconds. Strong verbal encouragement was provided to ensure maximal effort. The highest force recorded during the trials was considered to be the MVIC for the quadriceps [47].

Gluteus medius MVIC measurement: Participants lay on their side, with the test leg on top. The hip was positioned in neutral alignment. A strap was placed just above the knee to provide resistance. They were instructed to perform a maximal isometric hip abduction against the strap for five seconds. Strong verbal encouragement was provided to ensure maximal effort. The highest force recorded during the trials was considered to be the MVIC for the gluteus medius [48].

By normalizing electromyographic signals to these MVIC values, we accounted for individual variations in muscle strength, facilitating accurate comparisons of muscle activation levels across different participants and tasks [49].

### 2.6. Statistical Analysis

All statistical analyses were performed using IBM SPSS v26.0 (IBM Corp., Armonk, NY, USA). The normality of data distributions was assessed using the Shapiro–Wilk test. Depending on the data distribution, either independent *t*-tests (for normally distributed data) or Mann–Whitney U tests (for non-normally distributed data) were used to compare QASLSs and muscle activation levels between the ACL and control groups.

A *p*-value of less than 0.05 was considered to be statistically significant. We calculated the effect size for each result, including its ranges and significance. For parametric tests, we used Cohen’s d (small = 0.2, medium = 0.5, large = 0.8), while for non-parametric tests, we applied the appropriate effect size (ES) measure (small = 0.1, medium = 0.3, large = 0.5).

## 3. Results

### 3.1. Participant Characteristics

A total of 16 participants were included in the study, with 9 individuals in the ACL reconstruction group and 7 in the control group. The mean age in the ACL group was 26.3 ± 4.2 years, while in the control group, it was 25.8 ± 3.9 years. There were no significant differences in height (ACL: 1.78 ± 0.07 m, control: 1.76 ± 0.06 m; *p* > 0.05) or body mass (ACL: 74.5 ± 6.8 kg, control: 72.3 ± 5.9 kg; *p* > 0.05) between the groups (Table 1).

### 3.2. Neuromuscular Control Assessment

**Figure 3:**
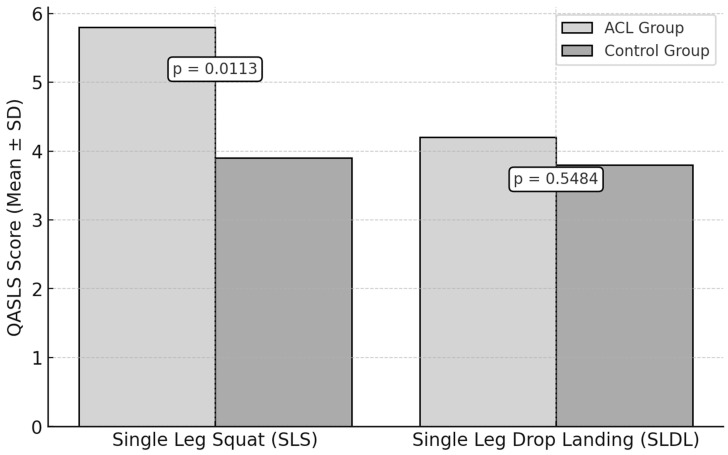
Neuromuscular Control Assessment: QASLSs in ACL vs. Control Groups.

The QASLS was used to evaluate movement quality during the SLS test and the SLDL test (Figure 3).

For the SLS test, the ACL group had a mean QASLS of 5.8 ± 1.4, while the control group scored 3.9 ± 1.2. A statistically significant difference was observed (*p* = 0.0113, ES = 1.44).

For the SLDL test, the ACL group obtained a mean QASLS of 4.2 ± 1.5, compared to 3.8 ± 1.1 in the control group. No statistically significant difference was found (*p* = 0.5484, ES = 0.30).

### 3.3. Gluteus Medius Activation

**Figure 4:**
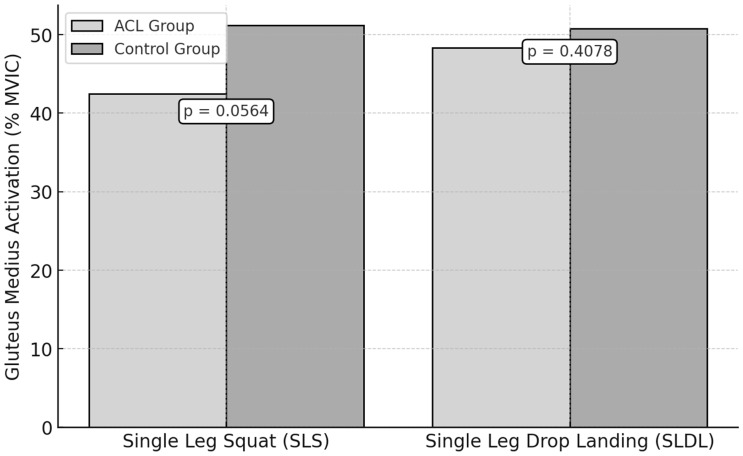
Gluteus Medius Activation During Functional Tests in ACL vs. Control Groups.

During the SLS test, the ACL group exhibited a mean peak activation of the gluteus medius of 42.7 ± 8.9% MVIC, while the control group recorded a 51.3 ± 7.6% MVIC (Figure 4). However, this difference was not statistically significant (*p* = 0.0564, ES = 1.03).

For the SLDL test, the mean peak activation was 47.1 ± 9.2% MVIC in the ACL group and 50.8 ± 8.1% MVIC in the control group. No significant difference was observed (*p* = 0.4078, ES = 0.42).

### 3.4. Vastus Medialis Activation

For the SLS test, the vastus medialis peak activation was 59.2 ± 10.5% MVIC in the ACL group and 50.1 ± 9.7% MVIC in the control group. However, this was not statistically significant (*p* = 0.095, ES = 0.90).

**Figure 5:**
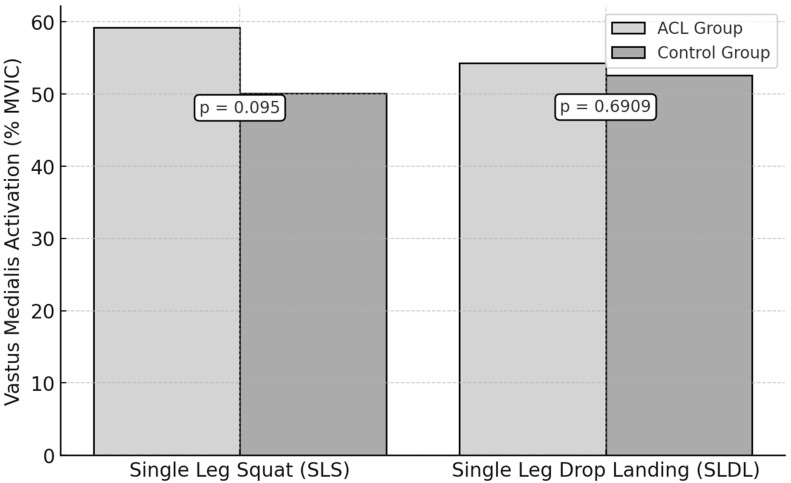
Vastus Medialis Activation During Functional Tests in ACL vs. Control Groups.

During the SLDL test, the ACL group showed a peak activation of 54.3 ± 8.8% MVIC, while the control group recorded a 52.6 ± 7.9% MVIC (Figure 5). No statistically significant difference was observed (*p* = 0.6909, ES = 0.20).

## 4. Discussion

This study investigated neuromuscular control and muscle activation patterns in individuals following ACL reconstruction, compared to healthy controls. The results indicate that neuromuscular control deficits persist post-ACL-reconstruction, as demonstrated by significantly higher QASLSs in the ACL group during the SLS test. However, no significant difference was observed during the SLDL test, suggesting that neuromuscular control deficits may be more apparent in controlled, slow movements rather than dynamic landing tasks. Additionally, gluteus medius activation was lower in the ACL group during the SLS test but not during the SLDL test, while vastus medialis activation showed a trend toward being higher in the ACL group, though it did not reach statistical significance.

These findings support existing research indicating that post-ACL neuromuscular impairments extend beyond knee function, affecting proximal hip stability and lower-limb coordination [12,30]. The implications of these results are particularly relevant in the context of injury prevention and rehabilitation, as neuromuscular control deficits have been strongly linked to increased risk of reinjury and altered movement mechanics [6,36,50].

### 4.1. Neuromuscular Control and QASLSs

A key finding of this study included the higher QASLSs observed in the ACL group during the SLS test, indicating poorer movement quality and neuromuscular control deficits. The significantly higher QASLSs in the ACL group during the SLS test, coupled with a large effect size (ES = 1.44), reinforce the presence of persistent neuromuscular deficits. These results align with prior studies reporting persistent biomechanical alterations in ACL-reconstructed individuals, particularly related to dynamic knee valgus, trunk instability, and altered weight distribution [30,34,51,52]. The SLS test is commonly used in clinical and research settings to assess lower-limb control, and our findings confirm its sensitivity in detecting neuromuscular control deficits in ACL-reconstructed individuals.

In contrast, the SLDL test showed no significant differences, with only a small effect size (ES = 0.30), suggesting that controlled squat movements may be more sensitive for detecting post-ACL neuromuscular impairments than dynamic landing tasks. This could be explained by the nature of dynamic landing tasks, where pre-programmed neuromuscular activation strategies might temporarily compensate for underlying deficits [53,54,55]. Previous research suggests that landing movements often elicit a more coordinated co-activation of hip, knee, and ankle muscles, which may mask subtle neuromuscular impairments [56]. Additionally, visual and vestibular inputs may play a role in optimizing movement quality during dynamic tasks, whereas controlled movements like squats rely more on intrinsic neuromuscular coordination [22]. These findings suggest that single-leg squat tests may be more effective in assessing neuromuscular deficits in ACL-reconstructed individuals compared to drop landing tests.

### 4.2. Gluteus Medius Activation and Hip Stability

The reduced gluteus medius activation observed in the ACL group during the SLS test highlights the role of the hip musculature in lower-limb stability post-ACL-reconstruction. The gluteus medius plays a crucial role in frontal plane stability, particularly in controlling hip adduction and internal rotation, which in turn influences knee valgus angles [14]. Deficits in gluteus medius function have been widely associated with increased ACL injury risk and altered lower-limb biomechanics [18].

These findings suggest that hip muscle weakness or neuromuscular inhibition could contribute to compensatory knee valgus movement patterns, which have been linked to higher ACL reinjury rates [6]. Previous research indicates that post-ACL neuromuscular inhibition often extends beyond the knee joint, affecting hip and core stabilization mechanisms [30]. This reinforces the importance of hip strengthening and neuromuscular training programs in ACL rehabilitation to address proximal control deficits [22].

Interestingly, gluteus medius activation did not differ significantly between groups in the SLDL test. One possible explanation is that landing tasks require greater whole-body coordination, thereby distributing stabilization demands across multiple muscle groups [57]. Additionally, compensatory muscle activation strategies, such as increased trunk engagement or ankle stabilization, may have minimized observable differences in gluteus medius activation during landing [52]. This further highlights the importance of selecting task-specific assessments when evaluating post-ACL neuromuscular impairments.

### 4.3. Vastus Medialis Activation and Quadriceps Function

The trend toward higher vastus medialis activation in the ACL group during the SLS test suggests potential altered quadriceps recruitment patterns post-ACL-reconstruction. While this difference was not statistically significant, the finding aligns with research showing that quadriceps neuromuscular control is often affected following ACL injury [9,12,58,59]. The vastus medialis plays a key role in knee joint stability and patellar tracking, and altered activation patterns could indicate compensatory mechanisms aimed at stabilizing the knee joint during unilateral weight-bearing tasks.

The observed increase in vastus medialis activation may reflect increased reliance on the quadriceps to compensate for residual deficits in knee stability [12]. This compensatory strategy has been reported in previous studies, particularly in individuals who exhibit persistent quadriceps inhibition post-reconstruction [9,60,61]. However, the lack of a significant difference in vastus medialis activation during the SLDL test suggests that landing movements may elicit a more global neuromuscular response, reducing the relative contribution of the vastus medialis [56].

### 4.4. Comparison with Existing Methods for Assessing Neuromuscular Control

Common approaches include the test–retest reliability analysis, which quantifies variability in performance over repeated trials, and joint-position sense assessments, which measure proprioceptive deficits by evaluating joint-angle reproduction errors [6,26]. While these approaches provide valuable insights into neuromuscular function, our study utilized the QASLS a validated tool designed to assess movement quality during weight-bearing tasks. The QASLS method is particularly relevant in the context of return-to-sport assessments, as it captures functional movement deficiencies that may not be evident in isolated proprioceptive or isokinetic strength tests [10,13,30].

In addition to the QASLS, sEMG analysis allowed us to examine the real-time neuromuscular activation of key muscles involved in knee joint stabilization. Prior studies have used sEMG to assess muscle activation asymmetries post-ACL-reconstruction, revealing persistent deficits in quadriceps and hip musculature activation, even after clinical recovery [58]. Our findings are consistent with these studies, particularly regarding reduced gluteus medius activation, which supports the growing consensus that hip muscle function is crucial for ACL injury prevention and rehabilitation [18].

### 4.5. Importance of Time Since Surgery: Comparing with Studies on Return to Sport

One of the unique aspects of our study is the post-operative time point at which assessments were conducted. Our participants were, on average, 14 months post-surgery, a period that is often considered crucial for return-to-sport readiness [37]. The reviewer correctly highlighted that this timeframe could be seen as an advantage, as it allows for a more realistic evaluation of long-term neuromuscular deficits compared to studies conducted in the early post-operative phase (6–9 months post-ACL-reconstruction) [11].

Studies tracking athletes who successfully return to sport after an ACL reconstruction indicate that neuromuscular control deficits can persist for over a year post-operatively, even in individuals who meet traditional strength and functional criteria for return to play [6,29,54]. Comparing our results with such studies underscores the importance of long-term monitoring and neuromuscular training interventions beyond the typical rehabilitation timeframe. Given the high reinjury rates observed in athletes who return to play with residual movement deficits, these findings reinforce the need for extended rehabilitation focusing on movement quality rather than isolated strength metrics [26].

### 4.6. Future Directions: Bridging the Gap Between Research and Clinical Practice

Moving forward, future research should explore longitudinal assessments of neuromuscular function in ACL-reconstructed individuals, tracking changes in movement quality and muscle activation patterns over time. Additionally, combining QASLSs with 3D motion capture and force-plate analysis could provide a more comprehensive evaluation of biomechanical adaptations post-ACL-reconstruction [53]. Finally, integrating psychological-readiness assessments alongside neuromuscular evaluations could offer a more holistic approach to return-to-sport decision-making, addressing both the biomechanical and cognitive factors influencing movement patterns [29].

In summary, this study highlights persistent neuromuscular deficits post-ACL-reconstruction, particularly during controlled, weight-bearing tasks such as the single-leg squat. By incorporating comparisons with previous studies, emphasizing the clinical relevance of our post-operative timeframe, and considering alternative neuromuscular assessment methods, we provide a broader perspective on ACL rehabilitation and return-to-sport strategies. These insights reinforce the importance of targeted neuromuscular training, with a focus on hip stabilization and quadriceps motor control, to optimize recovery outcomes and reduce the risk of reinjury.

## 5. Conclusions

This study highlights persistent neuromuscular control deficits in ACL-reconstructed individuals, particularly during single-leg squat tasks, where poorer movement quality and reduced gluteus medius activation were observed. These findings emphasize the need for targeted rehabilitation strategies, focusing on hip stabilization and quadriceps motor control to improve joint stability and movement efficiency. The results reinforce the importance of functional assessments, as single-leg squat tests proved more effective in identifying neuromuscular impairments than dynamic landing tasks. Despite limitations such as the small sample size and cross-sectional design, this study provides valuable insights into post-ACL recovery and rehabilitation strategies.

### 5.1. Limits and Perspectives

#### 5.1.1. Study Limitations

While this study provides valuable insights into neuromuscular control deficits and muscle activation patterns following ACL reconstruction, several limitations should be acknowledged. One of the primary limitations is the small sample size (n = 16), which may limit the generalizability of the findings. A larger sample would increase the statistical power and improve the ability to detect subtle neuromuscular differences between ACL-reconstructed individuals and healthy controls. Future studies should include a larger cohort to confirm these findings and explore potential subgroup variations based on factors such as the time since surgery, type of graft used, and level of physical activity.

Another limitation is the use of sEMG to assess muscle activation patterns. While sEMG is a well-established technique for neuromuscular assessment, it has inherent limitations, such as the potential for cross-talk between adjacent muscles and variability in electrode placement [49]. Future studies should consider using high-density sEMG or intramuscular EMG for a more precise analysis of individual motor unit recruitment and muscle activation timing.

Additionally, this study only focused on two functional tasks (SLS and SLDL) to assess neuromuscular control. While these tests are widely used in ACL rehabilitation, other movement tasks, such as cutting maneuvers, deceleration tasks, or agility drills, may provide additional insights into sport-specific movement adaptations [37]. Future research should incorporate a broader range of functional assessments to better capture task-dependent neuromuscular deficits.

Another important consideration is that this study did not account for psychological factors, such as the fear of reinjury or movement apprehension, which have been shown to influence movement patterns and neuromuscular control in ACL-reconstructed individuals [29,62]. Integrating psychological assessments, such as the ACL Return to Sport after Injury scale, could help elucidate the interaction between neuromuscular and psychological factors in post-ACL recovery.

One of the limitations of this study is that men and women were included together in the analysis. However, it is widely recognized that significant sex-based differences exist in ACL anatomy, biomechanics, and neuromuscular control. Women generally exhibit a higher risk of ACL injury due to factors such as increased ligament laxity, differences in lower-limb alignment, hormonal influences, and altered neuromuscular activation patterns. Additionally, previous research has suggested that female athletes may demonstrate greater knee valgus angles and reduced hamstring activation during landing and cutting tasks, which can influence post-reconstruction movement mechanics. Future studies should consider conducting separate analyses for men and women to better understand sex-specific neuromuscular adaptations following ACL reconstruction and to develop more tailored rehabilitation strategies.

Finally, the study design was cross-sectional, meaning it provides a snapshot of neuromuscular control at a single point in time. A longitudinal approach would allow for a better understanding of how neuromuscular control evolves throughout rehabilitation and whether specific interventions lead to sustained improvements in movement quality and muscle activation.

#### 5.1.2. Perspectives for Future Research and Clinical Applications

Building on these findings, several perspectives can be proposed for future research and clinical applications. First, longitudinal studies should be conducted to track neuromuscular adaptations over time, from the early phases of rehabilitation to the return-to-sport stage. This would help to identify critical time points when neuromuscular deficits are most pronounced and determine the effectiveness of different rehabilitation strategies [30]. Second, integrating 3D motion-capture systems and kinematic analysis could provide a more comprehensive evaluation of joint loading and movement strategies. Combining these methods with force-plate analysis would offer a detailed biomechanical profile of ACL-reconstructed individuals, allowing clinicians to design personalized rehabilitation programs [53,54,63,64].

Additionally, there is growing interest in individualized rehabilitation protocols based on neuromuscular profiling. Future studies could explore machine learning models to predict injury risk and rehabilitation success based on movement patterns, EMG data, and clinical assessments. These models could aid in developing precision rehabilitation programs tailored to each patient’s neuromuscular profile [26,65,66,67].

From a clinical perspective, the findings reinforce the importance of targeted neuromuscular training, particularly emphasizing hip strengthening exercises and quadriceps activation strategies. Incorporating proprioceptive training, perturbation-based exercises, and external feedback mechanisms could help to optimize motor learning and movement control in ACL-reconstructed individuals [29,62,68].

Finally, future research should explore the interaction between neuromuscular control and psychological readiness for returning to sport. Given that fear of reinjury can influence movement mechanics, interventions that address both neuromuscular and psychological factors may improve functional outcomes and long-term knee health [69].

## Figures and Tables

**Table 1:** Participant Characteristics.

Characteristic	ACL Group (n = 9)	Control Group (n = 7)	*p*-Value
Age (years)	26.3 ± 4.2	25.8 ± 3.9	0.728
Height (m)	1.78 ± 0.07	1.76 ± 0.06	0.543
Body mass (kg)	74.5 ± 6.8	72.3 ± 5.9	0.611
BMI (kg/m^2^)	23.5 ± 2.1	22.9 ± 1.9	0.502
Sex (M/F)	6/3	5/2	0.800
Time since ACL reconstruction (months)	5.70 ± 0.26	N/A	N/A
Injury mechanism			
- Indirect contact	35.20%	N/A	N/A
- Direct contact	0%	N/A	N/A
- Non-contact	64.80%	N/A	N/A
Associated lesions			
- None (isolated ACL)	60.50%	N/A	N/A
- Meniscectomy	18.40%	N/A	N/A
- Meniscus repair	12.30%	N/A	N/A
- Other lesions (cartilage, MCL, LCL)	8.80%	N/A	N/A
Quadriceps MVIC (Nm)	160.2 ± 13.8	187.6 ± 11.4	0.015
Gluteus medius MVIC (Nm)	85.4 ± 9.2	102.3 ± 8.7	0.021

Note: ACL; anterior cruciate ligament, SD; standard deviation, m; meters, kg; kilograms, M; male, F; female, MVIC; Maximal Voluntary Isometric Contraction, Nm; Newtonmeter, MCL; medial collateral ligament, LCL; lateral collateral ligament.

## Data Availability

The data presented in this study are available on request from the corresponding author.
